# A microfluidic chamber-based approach to map the shear moduli of vascular cells and other soft materials

**DOI:** 10.1038/s41598-017-02659-3

**Published:** 2017-05-23

**Authors:** Béla Suki, Yingying Hu, Naohiko Murata, Jasmin Imsirovic, Jarred R. Mondoñedo, Claudio L. N. de Oliveira, Niccole Schaible, Philip G. Allen, Ramaswamy Krishnan, Erzsébet Bartolák-Suki

**Affiliations:** 1 Department of Biomedical Engineering, Boston University, Boston, MA 02215 USA; 2 Center for Vascular Biology Research, Beth Israel Deaconess Medical Center, Harvard Medical School, Boston, MA 02215 USA

## Abstract

There is growing interest in quantifying vascular cell and tissue stiffness. Most measurement approaches, however, are incapable of assessing stiffness in the presence of physiological flows. We developed a microfluidic approach which allows measurement of shear modulus (*G*) during flow. The design included a chamber with glass windows allowing imaging with upright or inverted microscopes. Flow was controlled gravitationally to push culture media through the chamber. Fluorescent beads were conjugated to the sample surface and imaged before and during flow. Bead displacements were calculated from images and *G* was computed as the ratio of imposed shear stress to measured shear strain. Fluid-structure simulations showed that shear stress on the surface did not depend on sample stiffness. Our approach was verified by measuring the moduli of polyacrylamide gels of known stiffness. In human pulmonary microvascular endothelial cells, *G* was 20.4 ± 12 Pa and decreased by 20% and 22% with increasing shear stress and inhibition of non-muscle myosin II motors, respectively. The *G* showed a larger intra- than inter-cellular variability and it was mostly determined by the cytosol. Our shear modulus microscopy can thus map the spatial distribution of *G* of soft materials including gels, cells and tissues while allowing the visualization of microscopic structures such as the cytoskeleleton.

## Introduction

In many basic physiological processes^1^, such as the regulation of blood pressure^2^, and in diseases including cancer^3^, hypertension^4^, asthma^5^ and aging^6^, a governing role is ascribed to the mechanical properties of the constituent cells and tissues. The elasticity of cells^7–11^ and tissues^12–15^ in turn arise from the underlying structures whose maladaptation can have potent health consequences. For example, an increase in vascular wall stiffness, due to genetic determinants as well as the amount and organization of stiff wall components^16^, can precede hypertension and cardiovascular diseases^17^. Hence, there is growing interest in quantifying cell and tissue elasticity, especially in cardiovascular diseases.

Many experimental methods have been developed to measure cell and tissue stiffness. For example, shear wave elastography can estimate the macroscopic shear modulus (*G*) of the arterial wall *in vivo* from phase velocity measurements^18^. At the tissue level, the most widely used approach is to stretch a block of tissue uniaxially or biaxially and from the measurements of force and displacement, compute stresses and strains and the ratio of the changes in stress and strain define the modulus of the sample^1^. At the level of individual cells, elastic moduli can be determined using the atomic force microscopy (AFM) in indentation mode^7, 19^. Another method is to conjugate magnetic beads to the cell surface, apply magnetic twisting forces and from the measured bead displacement and the magnetic force, compute the shear stiffness^20, 21^. While these and other methods have provided a wealth of information on vascular tissue and cell elasticity, less attention has been paid to assessing *G* under physiological conditions such as blood flow in arteries and veins.

Recently, an experimental system was designed to specifically estimate endothelial whole cell shear stiffness under imposed physiological shear stresses^22^. The cytosol and the nucleus were imaged before and after flow and using image correlation analysis, average shear strain per cell was computed which allowed the calculation of an overall shear modulus of individual cells. However, this approach does not provide adequate spatial resolution to capture the wide distribution of intra- and inter-cellular stiffness along the apical surface of cells^7, 23^.

The purpose of this study was to develop a method to measure *G* of vascular cells and tissues under conditions mimicking blood flow. Since this requires measuring changes in both stress and strain, we would need to image the position on the surface of the sample of some markers such as fluorescent beads in the absence and presence of prescribed flow and hence shear stress. The central concept of our method is that the cell as a soft material under steady shear can be considered as a rigid surface. When exposed to a sudden change in shear stress, the cell, or any soft surface as a viscoelastic material, transiently changes its shape. However, once the transients die out and a new steady state is stabilized, the soft surface should act as a rigid surface independent of the prescribed shear stress. To confirm this, we examined the steady-state bead displacements on elastic surfaces with varying *G* under flow using fluid-structure numerical simulations. We then designed and tested a microfluidic chamber to impose well-defined shear stresses on the surface of gels, cells or tissue. In order to estimate *G*, we measured the displacement of fluorescent beads conjugated to the surface of polyacrylamide (PAA) gels, vascular cells and tissue and computed the local *G* as the ratio of imposed shear stress and measured shear strain.

## Results

### Computational simulations

**Figure 1:**
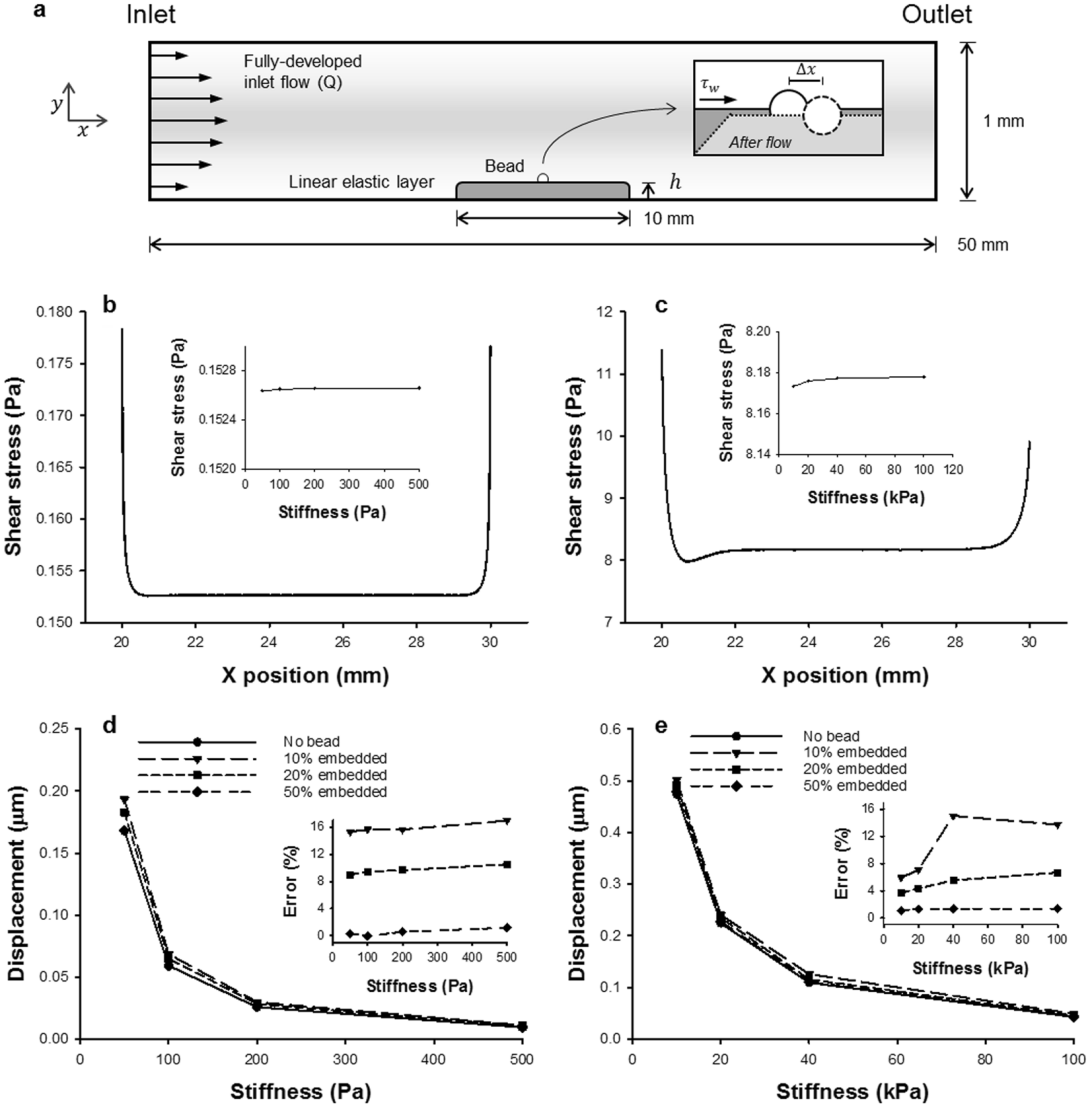
Numerical simulations of wall shear stress (*τ*_*w*_) and displacement (Δ*x*) for a linearly elastic layer representing a cell or tissue layer. A schematic of the 2D flow chamber simulations is shown in (**a**). Here, a linearly elastic solid was attached to the bottom of the chamber and exposed to a fully-developed flow at the inlet position. A rigid bead (diameter of 1 or 4 μm for cell or tissue, respectively) was attached at the midpoint of the elastic layer and embedded to 10, 20, or 50% of its diameter. Panels (b) and (d) correspond to simulations for a cell layer with Young’s moduli ranging between 50 and 500 Pa, whereas panels (c) and (e) correspond to simulations for a tissue layer with higher stiffness ranging between 10 and 100 kPa. In both cases, *τ*_*w*_ (panels b and c) was observed to be independent of stiffness and relatively constant across the length of the elastic solid (with the exception of expected increases at the boundaries). The insets demonstrate virtually no change in *τ*_*w*_ at the midpoint of the solid as a function of the stiffness. Furthermore, Δ*x* (panels d and e) at the midpoint of the elastic solid was shown to decrease with increases in stiffness for both the cell and tissue simulations, as would be expected. The insets demonstrate that although the error in Δ*x* using the center of the bead compared to no bead can reach ~16% when the bead is only marginally embedded (e.g., 10%), the error quickly approaches zero as the embedding increases.

Figure 1 shows the wall shear stress *τ*_*w*_ and horizontal displacement Δ*x* of elastic layers, mimicking cells and tissues, estimated from the microfluidic chamber computational simulations. For both cases, *τ*_*w*_ was relatively constant across the surface of the fluid-solid interface (panels b and c), with the exception of the strong boundary edge effects. The insets demonstrate that *τ*_*w*_ at the midpoint of the elastic solid was unaffected by the prescribed cell or tissue stiffness, which spanned an order of magnitude in each case. In contrast, the calculated Δ*x* decreased with increases in the prescribed modulus (panels d and e), as would be expected for stiffer cell and tissue layers. However, measuring displacement using the center of a bead attached to the elastic solid tended to overestimate the Δ*x* calculated for the same position on the cell or tissue layer. Nonetheless, as bead embedding approached 50% of the bead diameter, the error was nearly zero. Thus, our computational simulations indicate that shear-induced displacement along soft surfaces depends on the stiffness of the layer and can be estimated by tracking the movement of beads embedded on the surface.

### Imaging gels in the flow chamber

**Figure 2:**
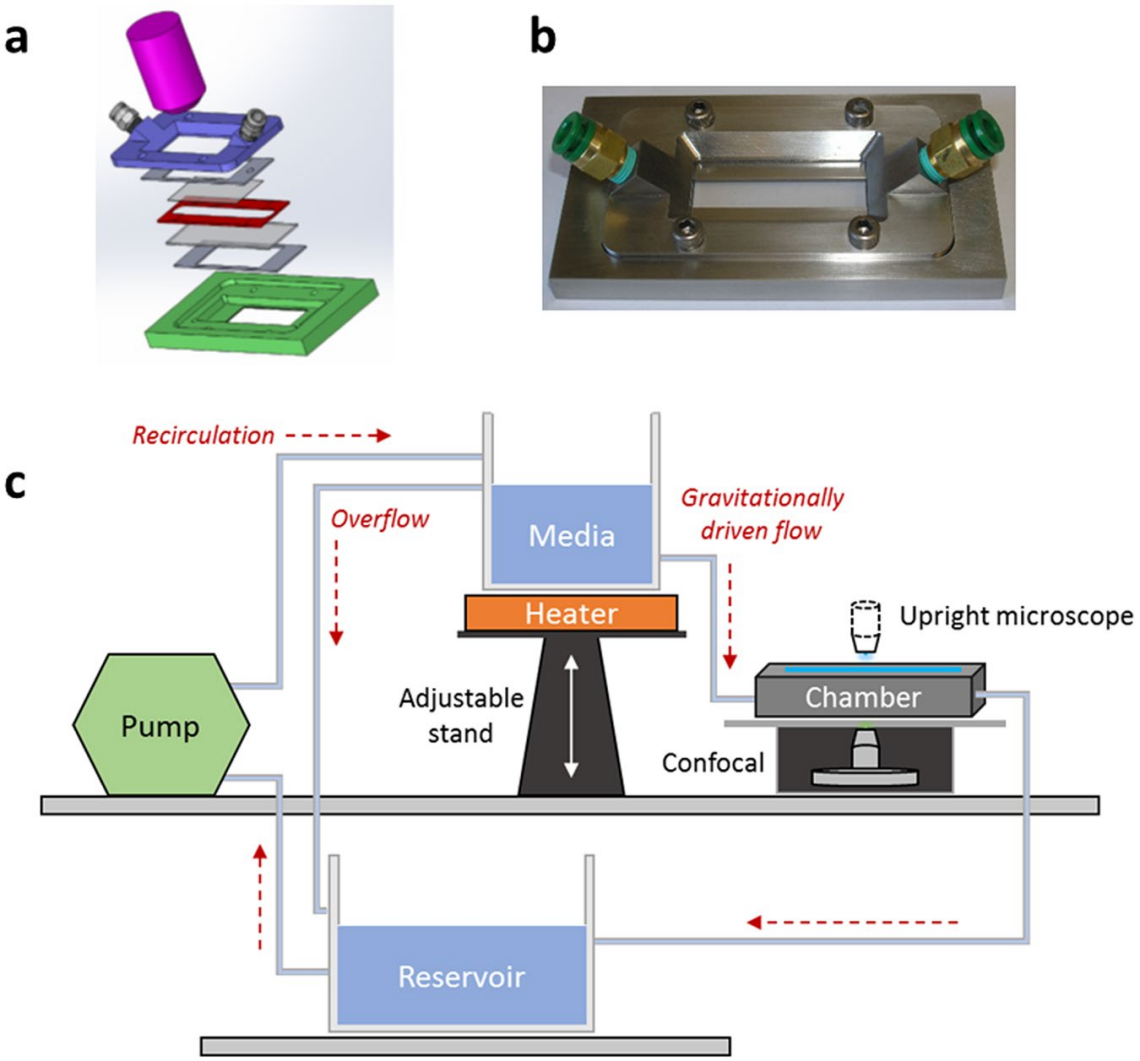
Design of the microfluidic flow chamber. (**a**) Schematic drawing of the layered structure of the microfluidic flow chamber. The green and blue pieces are the base and top, respectively, and the red piece is a spacer whose thickness can be varied between 0.1 and 1 mm. The gray layers below and above the spacer are cover slips. Cells can be grown on and tissue samples can be attached to either cover slip for imaging with a microscope (pink). (**b**) Actual picture of the final assembled device. The fluid inlet and outlet parts (green pieces) are connected to the chamber at an angle so as to reduce the change in flow direction as the fluid enters the chamber. (**c**) Schematic drawing of the flow circulation. Shear stress is generated in the chamber by a gravitationally driven flow by changing the elevation of the container containing heated media relative to the flow chamber. The outlet of the chamber is connected to a reservoir which is a buffer and from which a pump pushes fluid up into media. The overflow from the media to the reservoir maintains a constant height difference between media and the chamber.

**Figure 3:**
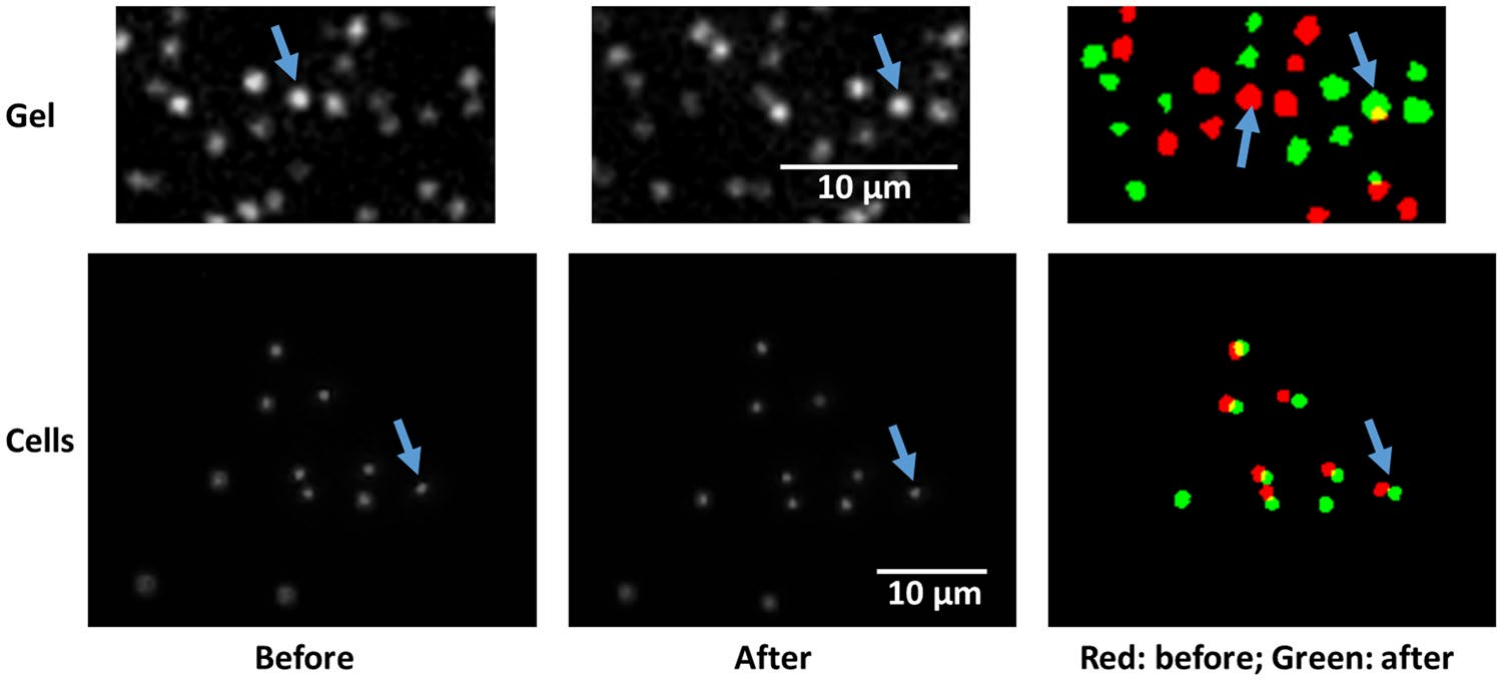
Examples of bead positions before and during flow. Bead positions and displacements due to shear stress on polyacrylamide gel (top row) and endothelial cells (bottom row). The left and middle columns correspond to bead positions before and after application of shear stress. The gel and the cells were exposed to a shear stress of 5.8 and 2.9 Pa, respectively. Notice the much larger displacements on the gel due to the ~100 times larger thickness of the sample. The right column demonstrates the identification of beads and their displacements. The red and green beads correspond to before and after shear stress conditions. The blue arrows follow a single bead through the images.

The design of the microfluidic flow chamber is described in the Methods and summarized in Fig. 2. The device allows imaging samples from both below and above using inverted and upright microscopes, respectively. Representative images of bead positions and their corresponding displacements are shown for both the PAA gel and a human pulmonary endothelial cell layer in Fig. 3. The mean and standard deviation (SD) of the thickness of the PAA gel, measured with confocal microscopy, was 300 ± 12 μm whereas that of the endothelial and vascular smooth muscle cells was 1.7 ± 0.6 μm and 3.8 ± 0.9 μm, respectively. Although the PAA gel was significantly stiffer, the displacements on the top of the gel were comparable due to the larger thickness.

To verify the microfluidic chamber’s ability to measure the shear modulus *G*, we used the PAA gel both in the chamber and in a uniaxial stretcher. The *G* of the PAA gel was estimated to be 1.76 ± 0.34 kPa independent of shear stress. The SD was obtained as the mean of the SD per image along the gel. The Young’s moduli of these gels, measured in the uniaxial stretcher, had corresponding values of 1.95 ± 0.31 kPa and there was no significant difference between the two sets of data (p = 0.279).

### Shear moduli of vascular cells and tissue

**Figure 4:**
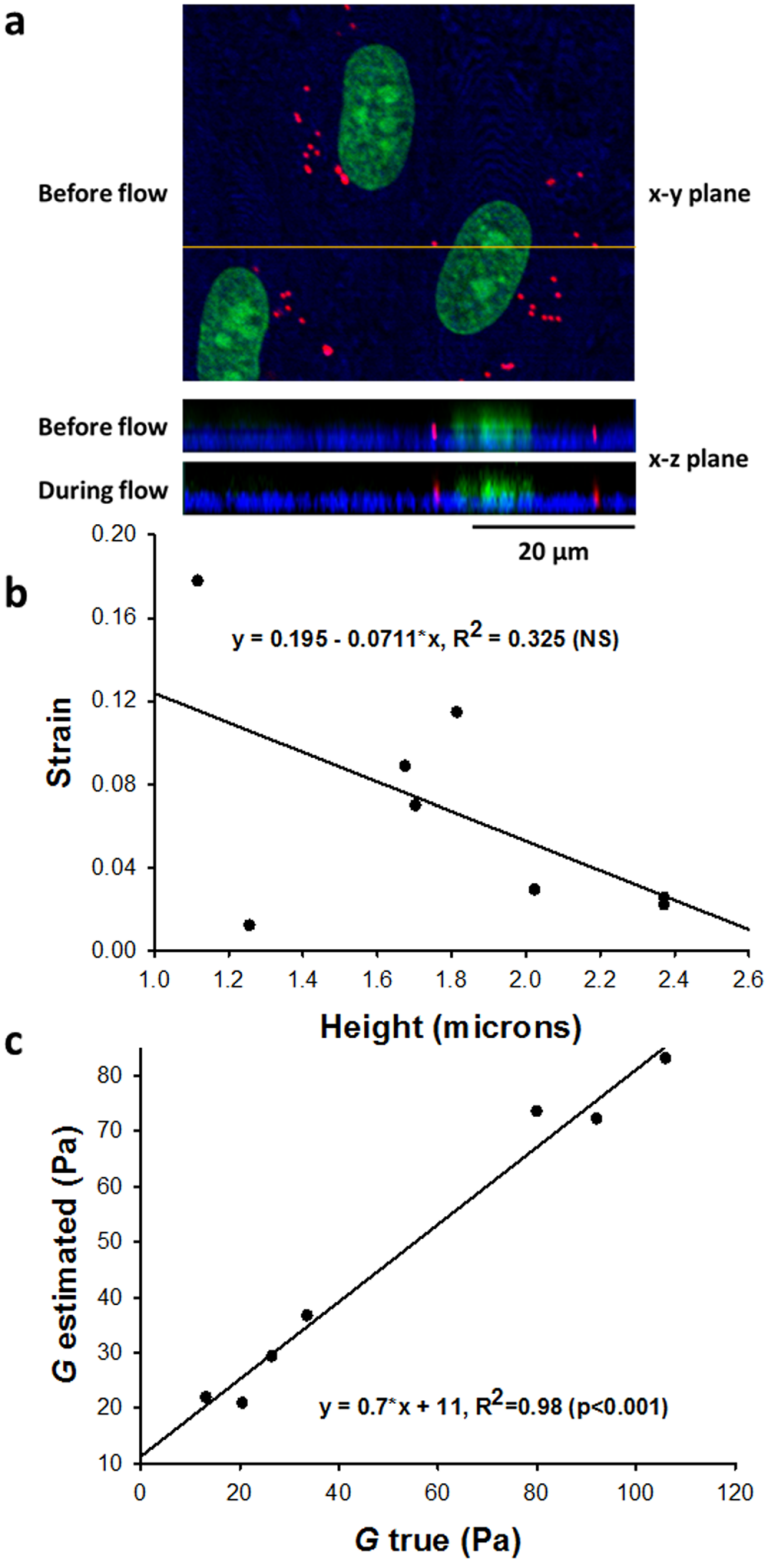
Estimation of endothelial cell shear modulus. (**a**) Confocal image of beads on top of the cells. The top image shows the glass (dark blue), nuclei (green) and beads (red) in the x-y plane. The middle image correspond to the x-z plane view of the same volume taken along the yellow line on the top image. Notice the two beads and the nucleus. The bottom image shows the same x-z plane view during shear flow in the x direction. Bead displacements in the x-z view are possible to detect. However, on the x-y plane the beads are circular and sub-pixel resolution displacements can be detected by computing the center of mass of the beads. The x-z view allows the measurement of bead distance from the top surface of the glass. (**b**) Shear strains obtained as the bead displacement due to flow divided by the height of the bead (Y position) as a function of height. There was not a significant correlation between shear strain and height. (**c**) Correlation between the shear modulus estimated by assuming a mean cell height for shear strain and “true” shear modulus using the actual height obtained from the confocal images.

Figure 4a shows endothelial cells inside the chamber with labeled nuclei (green) and red fluorescent beads on the cell surface. From the confocal images in the x-y plane, bead displacements can be obtained as in Fig. 3 whereas from the x-z plane, the distance between the bead and the glass can be estimated. Figure 4b suggest no correlation between bead height and bead displacement. Correspondingly, Fig. 4c demonstrates a very strong correlation between “true” *G* in which shear strains were obtained from individual bead heights and “estimated” *G* in which shear strains were computed based on the average height of all beads. These results suggest that only small and not systematic errors are introduced in the estimation of *G* when average height is used. This permits the evaluation of *G* without having to use a confocal microscope.

**Figure 5:**
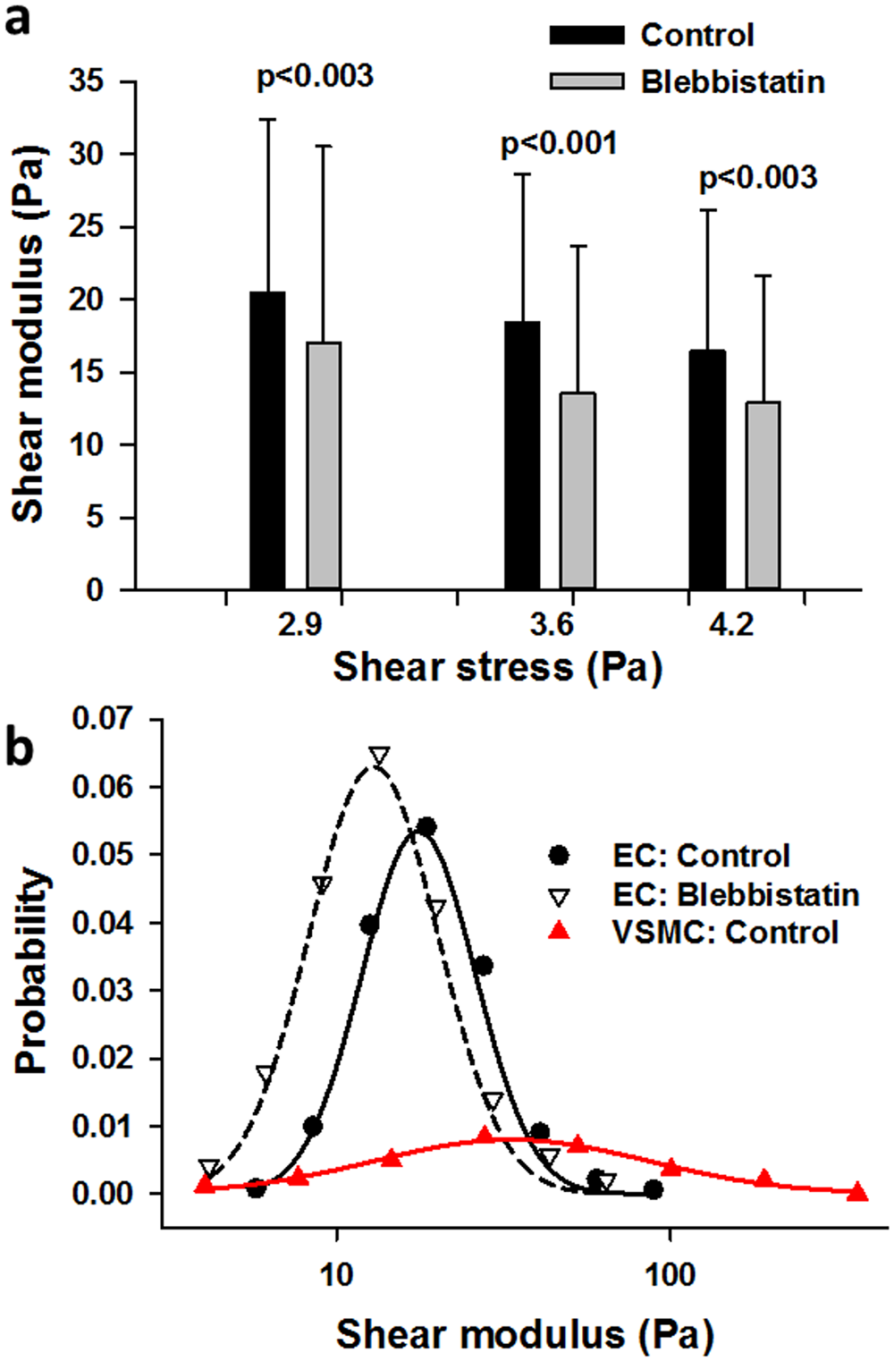
Shear moduli of endothelial and vascular smooth muscle cells. (**a**) The medians and interquartile ranges of shear moduli of >200 endothelial cells exposed to 3 levels of shear stresses before and after blebbistatin treatment. There is a small but highly significant (p < 0.001, repeated measure ANOVA) decrease of the shear modulus with increasing shear stress. Also, there is a strong difference between the moduli before and after blebbistatin at all 3 shear stress levels (ranksum tests). (**b**) The distributions of shear moduli (lumped together for all 3 shear stresses for endothelial cells) are similar to a lognormal distribution both in control and blebbistatin-treated endothelial cells. The solid and dashed black lines are fits of the lognormal distribution with means (22.2 and 17.3 Pa) and variances (3.7 and 3.8 Pa^2^) for the control and blebbistatin distributions, respectively. The read symbols correspond to the distribution of vascular smooth muscle cell (VSMC) moduli and the red solid line is the fit of the lognormal distribution with mean and variance of 89.3 Pa and 2.7 Pa^2^, respectively.

Shear modulus values in human pulmonary endothelial cells obtained at 3 physiological levels of shear stresses before and after treatment with blebbistatin for 30 min are summarized in Fig. 5a. Since blebbistatin inhibits myosin motors changing the tensile force on actin^24^, it is also expected to reduce *G* of the cell. Two findings are salient. First, increasing shear stress from 2.9 to 4.2 Pa produced a 20% and 24% decrease in *G* in control and blebbistatin treated cells, respectively. In both cases, this decrease was highly significant (p < 0.001). Next, blebbistatin significantly reduced *G* from control by an average of 22% (p < 0.003). Lumping all values separately in the control and blebbistatin groups suggests that the distribution of shear moduli is similar to a lognormal distribution whose mean decreased after blebbistatin treatment (Fig. 5b).

**Figure 6:**
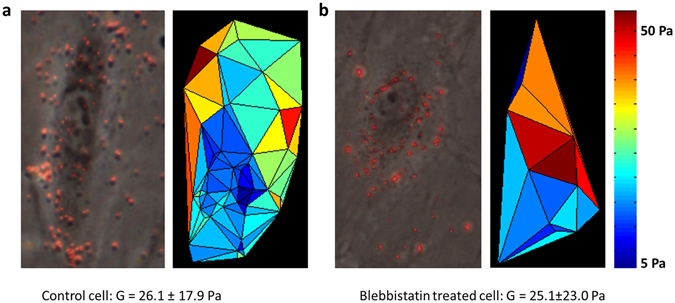
Example of shear modulus microscopy. Brightfield images with superimposed beads and the corresponding stiffness maps for a control cell (**a**) and a cell following blebbistatin treatment (**b**). The bead locations were used to create a Delaunay triangulation along the surface of the cell and each triangle is colored as the mean of the shear moduli of the three bead at the vertexes of the triangle.

In separate experiments performed with an imposed shear stress of 2.5 Pa, we also imaged cells in bright field which allowed us to identify the beads that belonged to a particular cell. An example of a control cell and a cell following blebbistatin treatment and the corresponding stiffness maps are illustrated in Fig. 6. In 10 control cells, we identified between 8 and 25 beads per cell whereas following blebbistatin treatment, in 14 cells we found between 5 and 27 beads per cell. The intracellular variability of *G* was 3.7 and 4.2 times higher than intercellular variability of *G* in control and blebbistatin-treated cells (p < 10^−7^). Additionally, both intra- and inter-cellular variabilities of *G* were higher after blebbistatin by 2.6 and 2.3 times (p < 10^−5^). The average coefficient of variation (*CV* = SD/mean) of *G* per cell was 44% and 69% in control and treated cells (p < 0.02).

**Figure 7:**
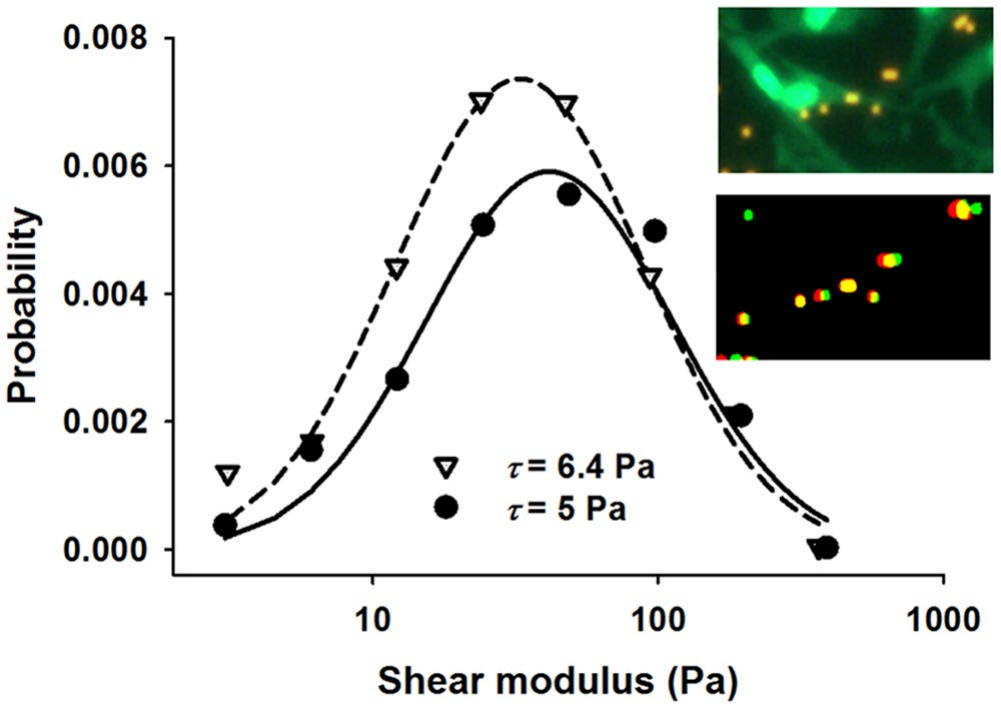
The distributions of shear moduli in vascular smooth muscle cells measured at two levels of shear stress (*τ*). Both distributions are similar to a lognormal distribution. The solid and dashed lines are fits of the lognormal distribution with means of 111.2 and 89.3 and Pa and variances of 2.7 and 2.7 Pa2 for the lower and higher shear stress cases, respectively. The top inset shows several vascular smooth muscle cells (green nuclear/cytosolic dye) and beads (red/yellow, 2.27 μm) before flow. The bottom inset shows only the beads before flow (red) and during flow (green, which partly becomes yellow if there is overlap).

To demonstrate that the method can be used with other cell types, we also measured *G* in vascular smooth muscle cells at two levels of shear stress, 5 and 6.4 Pa (Fig. 7). The median *G* slightly but significantly decreased with increasing shear stress from 58.5 to 44.5 Pa (p < 0.001). Additionally, these moduli are significantly higher than those of the endothelial cells (p < 0.0001) as can also be seen by comparing the corresponding distributions (Fig. 5b).

To further generalize the applicability of the method to vascular physiology, we implemented the technique in arterial wall segments. Images of the beads on the tissue surface were taken before and during shear flow and bead displacements were computed as in Fig. 3. The *G* of the endothelial cell layer on the inner surface of the aorta was estimated to be 97 Pa which is in the same order of magnitude as the moduli in cell culture since the tail of the distribution in Fig. 5b stretches beyond 90 Pa.

## Discussion

**Figure 8:**
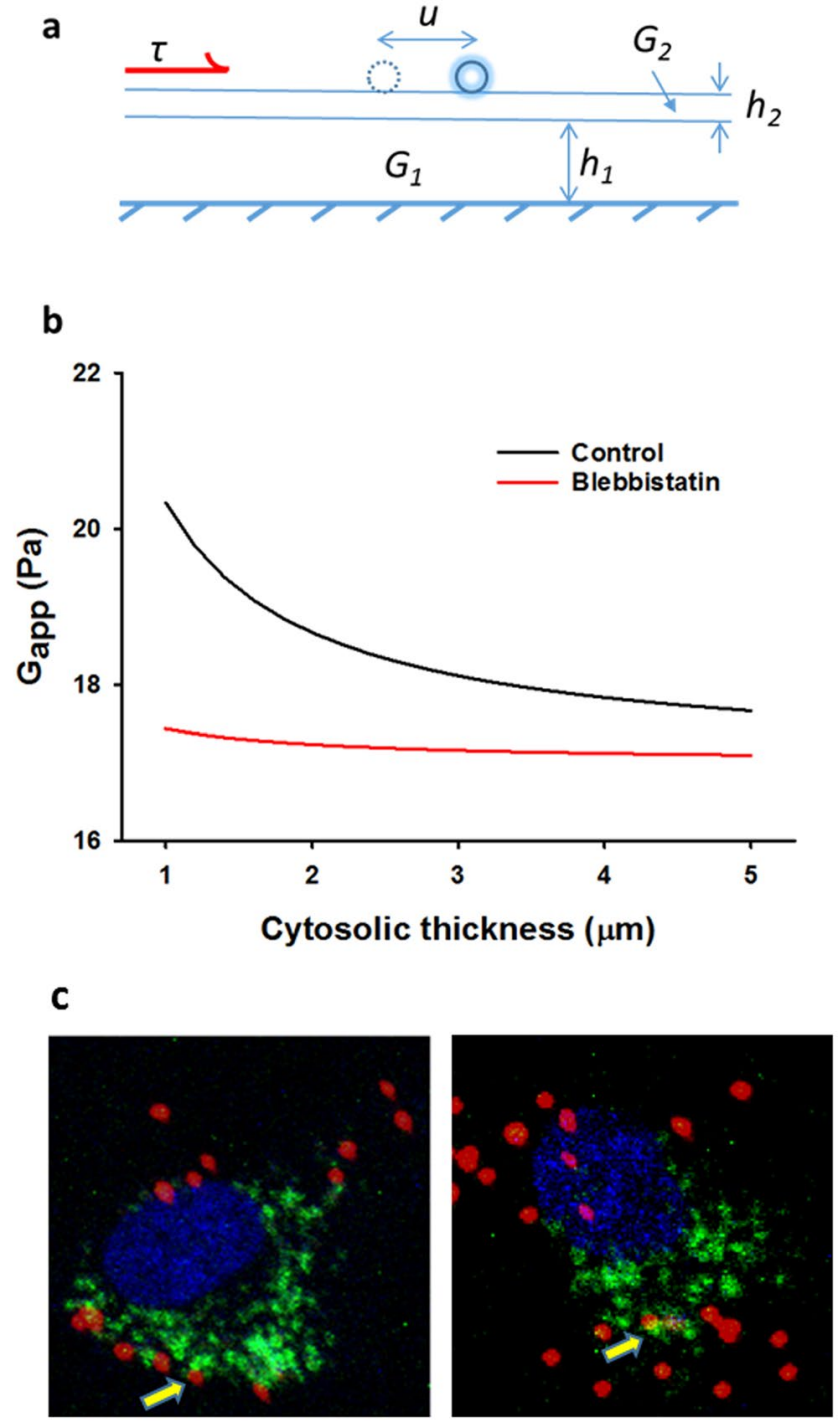
Theoretical interpretation of shear modulus and its experimental verification. (**a**) Modeling the cell as a two-layer elastic continuum exposed to shear stress at the top. The top layer represents the cell membrane and the actin cortex with modulus *G*_*2*_ and thickness *h*_*2*_. The bottom layer models the cytosol with modulus *G*_*1*_ and thickness *h*_*1*_. When the top surface is exposed to shear stress (*τ*), the bead moves to the right by a displacement *u*. (**b**) Simulation of apparent shear modulus (*G*_*app*_) of the model as a function of the thickness of the cytosolic layer in control cells (black line) using Eq. 3. The red line shows the simulated effect of blebbistatin by reducing *G*_*2*_ (see text for details). (**c**) Example images of a single cell labeled for nucleus (blue), beads (red) and lysosomes (green). The *G* was computed from the beads above lysosomes as well as from lysosomes where possible (yellow arrows).

In this study, we have introduced and tested a novel approach, we call shear modulus microscopy, which enables us to map the shear modulus distribution of cultured vascular cells, tissues and gels. The method can be extended to measure the shear moduli of any soft elastic material with a surface to which beads can be conjugated. Key advantages of the shear modulus microscopy are as follows. (1) The approach allows us to obtain a spatial distribution of shear moduli on the sample’s surface which can be important if the material is heterogeneous (see Fig. 6). Although conceptually different, the spatial map of *G* is similar to that produced by traction force microscopy^25–27^. (2) The measurement time is short and data to create a map of moduli can be obtained within 30 seconds. (3) In the case of biological materials, the experiments can be adjusted to expose cells or tissues to both physiological levels of shear stresses corresponding to *in vivo* conditions and pharmacological stimuli. (4) The chamber design also allows imaging the sample either from above, from below or both. The flexibility of the imaging setup can therefore be readily exploited to combine mechanical measurements with simultaneous visualization of intra-cellular structures. (5) The method may be extended to estimate intracellular shear moduli by injecting beads into the cytosol or using internal organelles such as the lysosomes (see below and Fig. 8).

There are several limitations to our method and the experimental system. First, the absolute value of the modulus critically and nonlinearly depends on the distance of the bead from the glass surface (Eq. 9 in Methods). Therefore, it was necessary to accurately measure the height of the PAA gel and the cell layer. However, fluctuations around the mean distance did not significantly affect the results (Fig. 4). These fluctuations may be due to changes in cell height which decreases from the center toward the periphery. The surface of the PAA gel also shows some roughness which together with the variable strength of bead binding might explain the variance of the PAA’s *G* along the surface. Second, to compute strain, we need to track the displacement of the top surface of the sample. The beads were on the top surface of the gel and may have been partially embedded in the cells. Thus, the beads also experiences both normal and shear stresses from the fluid flow which tend to rotate these beads. These factors were incorporated in our computations of the % error in displacement detected by bead motion relative to true surface displacement in Fig. 1d and e. These simulations provide evidence that the errors can reach 16% of the surface displacement if the embedding is less than 10% of the bead diameter. However, the confocal results in Fig. 4a suggest that this was not the case most likely due to the strong covalent bead attachment to the cell surface. Third, a key assumption of the method is that the shear stress exerted by the fluid flow on the sample surface is independent of *G* of the sample and constant over the region of interest. While this assumption is valid in the middle of the sample, we found strong variations in shear stress around the edges of the sample from the fluid-solid interaction simulations (Fig. 1b and c). Hence, imaging the beads should be limited to the middle portion of the sample. Another important point is that if the sample does not fully cover the bottom of the microfluidic chamber as in Fig. 1a, then the fluid will also exert significant normal stress on the front edge of the sample. Numerical simulations suggest that if the sample thickness is not small compared to the chamber height, then this normal stress will generate a lateral deformation of the sample that is somewhat similar to a simple shear of the top surface (not shown). In our case, the contribution of this effect to surface displacement was small. Additionally, *G* of the PAA gel obtained from the microfluidic measurements was in good agreement with that derived from uniaxial stretch.

In order to assess the possible errors in the estimation of *G*, we can consider *G* in Eq. 9 as a function of *h* and *d* and expand it into a Taylor series. Assuming that the covariance of *h* and *d* is zero, the variance of *G* (${\sigma }_{G}^{2}$) due to measurement errors is the sum of the variances of *h* and *d*, ${\sigma }_{h}^{2}$ and ${\sigma }_{d}^{2}$, respectively, each weighed by the square of the corresponding partial derivative of *G*. After some elementary calculations, the coefficient of variation *CV* of *G* (*CV*_*G*_) can be obtained as a function of the *CV* of *h* (*CV*_*h*_) and that of *d* (*CV*_*d*_) as follows:

1 $$C{V}_{G}=\,\sqrt{(1+\frac{2h}{H-h})C{V}_{h}^{2}+\,C{V}_{d}^{2}}$$

The displacement measurements used the beads’ center of gravity in the calculations. Hence, the *CV*_*d*_ is estimated to be small (<half a pixel relative to the size of the bead composed of >50 pixels), at most 0.05. For cells, *h* < 4 μm whereas *H* = 300 μm. Thus, the pre-factor of *CV*_*h*_ is close to 1 while *CV*_*h*_ is only 0.25 since most beads were not above the nucleus (Fig. 4a). These numbers provide a final estimate of 0.26 for *CV*_*G*_. This number is, however, less than half of the intracellular variability of *G* obtained from shear flow experiments. Furthermore, using mean *h* instead of actual *h* provided a good estimation of *G* (Fig. 4c). The *CV*_*h*_ for the gels was considerably less (<0.1) because of the even surface of the gels. Thus, we conclude that our method is able to provide accurate measurements of *G* on the surface of material with a stiffness ranging from under 1 Pa to at least 30 kPa, values that encompass most biological tissues.

In a recent study Ueki *et al*. proposed a method to measure *G* of whole cells^22^. The authors exposed endothelial cells to shear flow and used confocal imaging to measure cell deformation relative to the fixed bottom of their chamber. They employed image correlations to estimate the average shear strain of the entire cell from which they also estimated *G* utilizing the prescribed shear stress in the chamber based on bulk fluid mechanics. The reported moduli are 8–10 times larger than in our study. Several factors might reconcile this difference. First, they tested human umbilical vein endothelial cells as opposed to the pulmonary microvascular endothelial cells used in the current study. Second, our method of utilizing discrete beads provides a very accurate method of computing local shear strain which also gives a spatial map of *G* as opposed to the single cell bulk shear modulus value by Ueki *et al*.^22^. Finally, we paid careful attention to minimize any variation in normal stress before and during the flow measurement. Accordingly, the subsequent bead displacement is a function of imposed shear stress alone.

Other studies reported widely varying estimates of endothelial cell stiffness. Early magnetic twisting cytometry studies provided values between 20 and 80 Pa^21, 28^, comparable to our results. Sustained stretch of endothelial cells increased the stiffness^29^ which is opposite to what we find when we increased the shear stress (Fig. 5a). Furthermore, the modulus strongly depended on the ligand to which the beads were ligated^28^. Previous Atomic Force Microscopy (AFM) measurements of the Young’s modulus of human umbilical vein endothelial cells were 5 kPa with a corresponding *G* of 1.7 kPa^30^ which is significantly higher than our estimates. On the other hand, a magnetic tweezer-based microrheometry provided estimates of Young’s moduli around 400 Pa corresponding to a *G* of ~130 Pa^31^. Moreover, using bead tracking microrheology, Dangaria and Butler reported elastic moduli ranging between 0.1 and 10 Pa as a function of frequency^32^. These authors also found that exposure to 30 s of shear stress decreased the elastic moduli. Both the magnitudes and the shear stress dependence of their moduli are consistent with our findings.

In an attempt to interpret our results, we note that the AFM directly probes the cell membrane and the underlying subcortical actin. In contrast, in our setup, cells are globally exposed to shear stress and respond according to the local *G*. To better understand our measurement, we picture a system composed of two ideally elastic layers (Fig. 8a). The bottom layer has a thickness *h*_*1*_ and shear modulus *G*_*1*_, while the top layer is *h*_*2*_ thick with modulus *G*_*2*_. Using Eq. 6 with $\vec{v}$ = 0, assuming the normal stresses are also zero and the bottom layer is fixed while the top surface is exposed to a constant shear stress *τ*, we can easily solve the boundary value problem for the displacement of the top surface *u* as

2 $$u=\tau (\frac{{h}_{1}}{{G}_{1}}+\frac{{h}_{2}}{{G}_{2}})$$

Dividing *u* by (*h*_*1*_ + *h*_*2*_), we obtain the strain and dividing *τ* by this strain we obtain the apparent shear modulus *G*_*app*_ of the composite:

3 $${G}_{app}={(\frac{\alpha }{{G}_{1}}+\frac{1-\alpha }{{G}_{2}})}^{-1}$$

where *α* is the fractional thickness defined as *h*_*1*_/*(h*_*1*_ + *h*_*2*_ *)*. Assuming the cortical actin thickness is *h2* = 0.2 μm^33^, *G2* = 1 kPa and *G1* = 17 Pa, *G*_*app*_ can be estimated as a function of cytosolic height *h1*. As shown in Fig. 8b, for the measured cell heights, *G*_*app*_ varies approximately between 20.5 and 18 Pa. If we also assume blebbistatin reduces *G*_2_ to 20 Pa, then the simulations provide quantitative agreement with the data in Fig. 5a.

To test the validity of these predictions, we labeled lysosomes green in addition to the red beads in a complementary set of experiments. Cell nuclei, beads and lysosomes were imaged before and after flow using the confocal microscope. We then selected regions where distinct lysosome vesicles were found just under the fluorescent beads. From six such regions (see example in Fig. 8c), we determined *G* using the beads as well as the underlying lysosomes taken as near rigid particles. The lysosome-based *G* was 60.2 ± 3.6% of *G* computed using beads above the same lysosomes which is in good agreement with the notion of cytosolic contribution to *G*_*app*_ discussed above. Therefore, we conclude that our measurements are mostly determined by the cytosol and hence the likely contributors to *G*_*app*_ are the various motor proteins^34^ and possibly the loosely connected organelles in the cytosol.

The variability of *G* in cells was high reaching a *CV* of ~70% for endothelial cells (Fig. 5b) and ~100% for vascular smooth muscle cells (Fig. 7). On the other hand, the *CV* of *G* for the gels was less than 20%. Given the above analysis of *CV* (Equation 1) and the imaging results of Fig. 8c, we believe the high variability of *G* in cells is not an artifact; rather, it is a consequence of the heterogeneous intracellular structure. Furthermore, the spatial characterization of shear moduli across the pulmonary endothelial cell revealed that the intra-cellular variability of *G* is significantly higher than the inter-cellular variability (Fig. 5). An implication of this is that a single modulus measurement per cell may severely under- or over-estimate total cell stiffness particularly so because the distribution of the modulus is lognormal with a long tail. Furthermore, since blebbistatin inhibits non-muscle myosin II motors, the cortical actin tension within the cells should decrease following blebbistatin treatment which is supported by the fact that in our experiments the mean modulus decreased (Fig. 5a). However, unexpectedly, the variability of the modulus increased after blebbistatin treatment suggesting that subcortical actin tension, also called prestress, acts to homogenize cell mechanical properties. Therefore, the blebbistatin-induced break-up of the actin-myosin network may hinder efficient force transmission between different regions of the cell resulting in fragmented regions of highly variable local tension and stiffness. Alternatively, the lower inter-cellular shear moduli may be due to cell-cell mechanical interactions which appear to contribute to multicellular mechanical homeostasis as proposed recently^35^.

In terms of the experimental system, we note that while the gravitationally-driven flow is stable and free of vibrations that some pumps may generate, it is unable to produce cyclic or unidirectional but fluctuating shear stresses. Future studies could combine the stiffness measurement concept with precision controlled peristaltic pumps to achieve long-term continuous exposure of cells to shear stress.

In conclusion, we developed and validated a novel method to map regional shear moduli of soft samples including gels, cells, and tissues. Our results suggest that intra-cellular stiffness is significantly larger than inter-cellular stiffness and subcortical tension acts to homogenize regional distribution of shear moduli. As such, this methodology is well-suited to explore new mechanobiological relationships in cellular and tissue responses to flow.

## Methods

### Numerical computations

A 2D computational model of the microfluidic chamber was generated in the COMSOL Multiphysics^®^ (Version 4.3, COMSOL Inc.) software package to study the shear deformation of a linearly elastic solid, mimicking either a cell or tissue layer, during steady-state conditions. To model the relationship between fluid flow and solid deformation, the fluid-structure interaction physics module was used with the following governing equations:

4 $$\frac{\partial \rho }{\partial t}+\,\nabla (\rho \vec{v})=0$$

5 $$\rho \frac{\partial \vec{v}}{\partial t}+\,\rho (\vec{v}\cdot \nabla )\vec{v}=\,-\nabla p+\mu {\nabla }^{2}\vec{v}$$

6 $${\rho }_{s}\frac{{\partial }^{2}\vec{v}}{\partial {t}^{2}}=\,\nabla \cdot \sigma $$

where *ρ*, *μ*, and $\vec{v}$ are the density, viscosity, and velocity of the fluid, respectively; *p* is the pressure; *σ* and *ρ*_*s*_ are the stress tensor and the density of the elastic solid, respectively, and *t* is time. Equations (4) and (5) are the continuity and Navier-Stokes equations for an incompressible Newtonian viscous flow without gravity, while Eq. (6) describes the field equations for an elastic solid.

No slip boundary conditions were applied along the walls of the microfluidic chamber as well as the fluid-solid interface, while a rigid constraint connected the bottom of the elastic solid to the inner chamber wall. Flow at the inlet was considered to be fully-developed and thus, the velocity profile of the fluid was defined as follows:

7 $${v}_{x}=\frac{6Qy}{{H}^{3}}(H-y)\,{\rm{for}}\,0\le y\le H$$

where *Q* is the flow per unit width and *H* is the height of the chamber. Boundary conditions at the outlet were simply defined to be the pressure without viscous stress. The height and length of the chamber were assigned to be 1 and 50 mm, respectively. The elastic solid with rounded edges was centered on the midpoint of the chamber and assigned a length of 10 mm, while the height was either 10 or 150 µm for the cell or tissue layer, respectively. For all simulations, the fluid was assumed to be Newtonian with *ρ* of 1000 kg/m^3^ and *μ* of 0.001 Pa·s, the solid was modeled as a linearly elastic material with *ρ*_*s*_ of 960 kg/m^3^ and Poisson’s ratio (*ν*) of 0.49. A schematic of the simulation configuration is shown in Fig. 1a.

To determine the effect of the Young’s modulus (*Y*) on the horizontal shear displacement Δ*x* along the fluid-solid interface, simulations were performed using *Y* of 50, 100, 200, and 500 Pa with *Q* of 0.05 ml/s for the cell layer conditions, and *Y* of 10, 20, 40, 100 kPa with *Q* of 2.00 ml/s for the tissue layer conditions. For each simulation, the wall shear stress ${\tau }_{w}$ and Δ*x* were calculated as the output variables. In addition, simulations with a rigid bead (diameter of 1 or 4 µm) attached at the midpoint of the elastic solid were performed. These computations were used to compare Δ*x* calculated using the center of the bead with the actual Δ*x* calculated from the same point on the elastic solid layer without a bead. The same *Y* and *Q* were used as before, and the impact of bead embedding was characterized by defining its depth within the elastic solid as 10, 20, or 50% of the bead diameter for each case. Separate triangular meshes were applied to the fluid, solid, and bead domains individually optimized to ensure sufficient convergence over the different length scales of the problem. For all simulations, a fully coupled stationary MUMPS (Multifrontal Massively Parallel sparse direct Solver) was used. All computations were performed on a Lenovo^®^ W530 ThinkPad workstation.

### Device design

A microfluidic steel flow chamber was designed and manufactured with the assistance of the Scientific Instrument Facility at Boston University. The main design criteria were: (1) the total fluid flow resistance of the chamber should be such that the shear stress inside the channel can be conveniently set to physiological values using gravitationally driven flows; (2) a simple 2-dimensional (2D) plane flow should be maintained within the center of the chamber where imaging occurs; (3) the sample should be observable through either side of the chamber, top or bottom, with a microscope; and (4) chamber height should be flexible so as to accommodate thin cell layers as well as thicker vascular wall tissues. Accordingly, the final chamber design has a layered structure with transparent windows both on the top and bottom for imaging (Fig. 2a and b). The channel height can be varied between 0.1 and 1 mm using spacers and the width and length of the chamber are 10 and 75 mm, respectively.

Flow and shear stress was gravitationally driven in the chamber (Fig. 2c). The inlet of the chamber was connected via a stiff-walled plastic tube to a container which was filled with heated (37 °C) Endothelial Basal Medium-2 (EBM-2, Lonza) for endothelial cells or Dulbecco’s Modified Eagle Medium (DMEM) for vascular smooth muscle cells. For polyacrylamide (PAA) gels, phosphate buffered saline (PBS) was used at room temperature. The container with heated media was raised to a certain height such that the gravitationally driven flow generated the desired shear stress in the chamber. The outlet of the chamber was led through another plastic tube into a reservoir. A pump (Miniature gear pump, Cole-Parmer) was used to recirculate the fluid from the reservoir to media and an overflow channel guaranteed that the gravitational height was constant during the experiment. This arrangement isolated the pump from the chamber and hence no vibration from the pump disturbed imaging during flow.

The chamber inlet and outlet tube diameters and lengths were chosen so that the corresponding fluid flow resistances generated the desired mean pressure as well as shear stress inside the chamber. The pressure drop across the inlet and outlet was measured using a PC mountable differential pressure sensor (pxcpc-010wcdv, Omega Engineering, Inc.). The pressure data were collected through a NI DAQ board and processed in LabView. Flow at the outlet was measured by timing the collection of a fixed amount of fluid leaving the chamber.

A 2D laminar flow was generated in the chamber. The wall shear stress, *τ*_*w*_, on the sample surface can be varied from 0.1 to ~100 Pa by elevating the container relative to the chamber and/or changing *H*. Specifically, the shear stress on the sample is given by

8 $${\tau }_{w}=\frac{6\mu Q}{W{(H-h)}^{2}}$$

here, *Q* is the volumetric flow rate, *h* is the sample height and *W* is the chamber width. For tissue, *h* was between ~150 µm whereas for cells *h* was between 2 and 4 µm. The viscosity of PBS was taken to be 0.001 Pa·s whereas that of the cell culture media was 0.00074 Pa·s^22^.

### Cell/tissue preparation

All procedures were approved by the Institutional Animal Care and Use Committee of Boston University (Protocol # 12–031) and the experiments were performed in accordance with relevant guidelines and regulations. One male Wistar-Kyoto rat (age: 10 weeks) was anesthetized with a mixture solution of Xylazin^®^ (10 mg/kg) and Ketamin^®^ (90 mg/kg) and the thoracic aorta was harvested. An abdominal aortomy was performed to excise aorta. The aorta was then washed with PBS, cleaned from residual tissue on the outside, cut open and sectioned into pieces of about 2 × 5 mm. The edges of the sample were glued to a cover slip and labeled with beads for flow experiments.

Human pulmonary artery endothelial cells were purchased from Lonza Biologics Inc (Hopkinton, MA). Vascular smooth muscle cells were isolated from bovine aortae using the explant method as previously described^36^ and experiments were performed at the first passage. Cells were seeded in the middle section of collagen type I coated cover slips immersed in cell culture media and were allowed to attach for 24 h and subsequently labeled with beads described below.

### Fluorescent labeling

Carboxyl functionalized fluorescent beads were used to obtain stable covalently labeled tissue samples (5.0–5.9 µm) or cells (0.4–0.6 µm and 2.27 µm), with all bead types being high intensity Nile red particles (Spherotech Inc., Il, USA). The particles were diluted in serum-free cell culture media to 1.0 mg/ml before coating and the samples were incubated with the beads for 2 h. Lysosomes were labeled with the green fluorescence dye (Lysotracker Green, Molecular Probes) at 50 nM final concentration for 15 min and washed with media. Nuclei were labeled either blue with NucBlue Live ReadyProbes Reagent (ThermoFisher Scientific) or green with SYTO Green Fluorescent Nucleic Acid Stain which also labels the cytosol at 5 µM concentration (ThermoFisher Scientific). Following labeling, samples were mounted into the flow chamber for imaging. To preserve viability, cell and tissue samples were always kept in media throughout the process.

### Flow chamber Protocol

Once the chamber was assembled with the sample inside, an image of the beads in a given region was taken in the absence of flow. Under these conditions, the mean pressure inside the chamber was adjusted to be ~10 mmHg that corresponds to a typical pulmonary arterial pressure. The media container was then elevated by a pre-calculated amount to ensure that the mean pressure in the chamber during flow was the same as in the absence of flow. Next, the outlet of the chamber was opened and after the transients died out in 3–4 s, an image of the same region was taken again to assess the displacement of the beads affixed to the surface of the sample. This procedure was repeated for 2 and 3 flow magnitudes for vascular smooth muscle and endothelial cells, respectively. The flow was then stopped, another region suitable for imaging was selected and the procedure was repeated. In another set of experiments, cells were incubated with blebbistatin (5 μM) for 30 min before imaging in order to reduce cortical tension and stiffness of the cells.

### Gel experiments

We validated our approach using PAA gels with two different shear moduli. Briefly, a mixture containing Acrylamide/Bis-acrylamide (Bio-Rad, Hercules, CA) (5% Acrylamide and 0.1% Bis-acrylamide), ultrapure water, ammonia persulfate (0.5%), and TEMED (Bio-Rad, Hercules, CA) (0.05%) was polymerized between a glass coverslip pre-treated with NaOH and 3-aminopropyltrimethoxysilane and an untreated coverslip that had been incubated with red fluorescent beads (0.5 µm in diameter, Invitrogen, Carlsbad, CA). The process yielded gels with a final thickness of 100–400 μm^37^. The top surface of the gels was also ligated with the same fluorescent beads and the gel was placed into the flow chamber. Images of the beads were obtained before and after exposing the gels to 4–8 Pa shear stresses. The bottom beads were used to align the images before and during flow using cross-correlation whereas the bead displacements on the top surface were used to compute the shear strain associated with individual beads. From the ratio of the imposed shear stress and the measured shear strain, we computed *G*. This value was compared with traditional stress-strain measurements, described next.

Larger gel blocks prepared similarly as above were cut into pieces of 4 × 8 × 0.5 mm in dimension and assembled into a vertical uniaxial stretcher device^36^. Briefly, small metal plates were glued to the edges of the PAA sample with rigid wires. The wires were connected to a lever arm (model 300B, Aurora Scientific, Ontario, Canada) and a bidirectional, inductive-type force transducer (model LC-01, CSM Instruments, Switzerland). The sample was stretched to 20% strain at a rate of 0.5%/s and the force response was recorded. Stress was calculated by dividing the force with the cross sectional area and *Y* was obtained as the slope of the stress-strain curve at 5% strain. Optical imaging of the lateral contraction of the sample allowed us to estimate the Poisson’s ratio *ν* as the ratio of the lateral and longitudinal strains from which *G* of the gel was computed as *G* = 0.5*Y*/(1 + *ν*).

### Imaging

Bead displacements on the surface of the samples were imaged using an inverted (Nikon Eclipse TS100) or an upright microscope (Nikon Eclipse 50i). The fluorescent beads have EX/EM wavelength of 535/575 nm. A SPOT Insight^TM^ camera (SPOT Imaging Solutions) was used to capture images. To determine whether cell height influenced bead displacement, confocal fluorescence and reflectance imaging was carried out using an Olympus Fluo-view 1000 laser scanning confocal microscope. The 488 nm laser line was used to generate reflection from the glass-air or glass-sample interface, and the spectral selection was adjusted to pass the 488 nm laser light on to the detector. The red fluorescent beads were imaged by exciting with the 543 nm laser and detection in the range of 555–655 nm. For all experiments, we first identified markers such (e.g. scratches on the glass surface or beads at the bottom of gel) which provided an absolute reference. Corresponding images before and during flow were aligned either manually or via cross-correlations using these marks before further analysis. A custom MATLAB program was written to process the images. Using a pattern recognition, the program automatically identified the same beads at several levels of shear stresses. Changes in shear stress was obtained from fluid mechanics. Shear strains were computed as the ratio of bead displacement (*d*) to bead height (*h*) from the glass surface where *d* was the distance between the centroids of the bead before and during flow. The centroid was obtained from at least 30 pixels allowing subpixel resolution of *d*. Finally, *G* was estimated as the ratio of shear stress change to shear strain change as

9 $$G=\frac{6\mu Qh}{Wd{(H-h)}^{2}}$$

### Statistical analysis

Data are reported as means and SDs or medians and interquartile ranges. Changes in *G* due to shear stress were analyzed using non-parametric repeated measure ANOVA whereas the effect of blebbistatin was analyzed by ranksum test between control and treated cells separately at each shear stress level. Analysis of variability included a statistical model in which the main effect was blebbistatin and individual *G* values had random contribution from intra-cellular and inter-cellular responses which allows the separate estimation of the intra- and inter-cellular variance of *G*. Since the moduli data showed near lognormal distribution, a log transformation was used before comparing the variances using F test. Significance was accepted at the 0.05 level.
